# PW03-003 – Altered neutrophil function in PFAPA

**DOI:** 10.1186/1546-0096-11-S1-A229

**Published:** 2013-11-08

**Authors:** M Sundqvist, P Wekell, V Osla, J Bylund, K Christensson, K Sävman, A Fasth, S Berg, KL Brown, A Karlsson

**Affiliations:** 1 Dept Rheumatology and Inflammation Research, Gothenburg University, Gothenburg, Sweden; 2 Department of Pediatrics, NU-Hostpital Organization, Uddevalla, Sweden; 3 The Queen Silvia Children’s Hospital, Gothenburg, Germany; 4 Department of Pediatrics, Gothenburg University, Gothenburg, Sweden; 5 Department of Pediatrics, University of British Columbia, Vancouver, BC, Canada

## Introduction

The PFAPA syndrome is a non-mendelian autoinflammatory disease of unknown aetiology characterized by Periodic Fever, Aphthous stomatitis, Pharyngitis, and cervical Adenitis. In typical cases, attacks begin before the age of five and occur every 2-8 weeks, often with striking periodicity. We have previously profiled the blood cells and serum cytokine levels in a cohort study of patients with PFAPA (Brown et al, BMC Pediatric 2010). Since autoinflammatory diseases by definition are mediated by cells of the innate immune system, and the major innate immune cell, the neutrophil, is abundant in the circulation during PFAPA episodes we decided to investigate neutrophil function during the febrile and afebrile state in the same cohort.

## Objectives

The purpose of this study was to characterize functional features of neutrophils, during different phases PFAPA syndrome, including priming, production of reactive oxygen species (ROS), and apoptosis.

## Methods

Neutrophils (PMN) were isolated from blood collected from twelve PFAPA patients during febrile and afebrile phases (mean age 3.5 yrs), from 18 healthy control children (mean age 4.5 yrs), and from 4 febrile control children (mean age 4.8 yrs), with fever and abdominal pain due to infection or appendicitis. We assessed PMN apoptosis by annexin V staining, production of reactive oxygen species (ROS) by luminol/isoluminol-amplified chemiluminescence, MPO quantity by ELISA, and degranulation/priming as expression of CD11b and L-selectin by flow cytometry.

## Results

We found a significantly increased intracellular (ic) production of ROS in response to PMA in PMN during the febrile phase, as compared to cells from the afebrile phase. No differences were found in MPO quantity between the samples indicating that the increase in icROS was due to increased NADPH-oxidase activation. We also found decreased spontaneous apoptosis in PMN during the febrile phase of PFAPA, while apoptosis was instead increased during the afebrile phase. Increased expression of CD11b indicated that the PMN were slightly primed during the febrile phase, supported by increased oxidative response to galectin-3, a priming-dependent process. Preliminary results indicate that the latter are not general features of PMN in children with fever, as the febrile control children were significantly lower in degranulation as well as galectin-3-induced ROS production.

## Conclusion

Alterations in PMN function, e.g., icROS-production, priming and spontaneous apoptosis, may give mechanistic clues to the so-far undefined etiology of the PFAPA syndrome.

## Disclosure of interest

None declared

